# The impact of the two-year COVID-19 pandemic on hospital admission and readmissions of children and adolescents because of mental health problems

**DOI:** 10.3389/fpubh.2023.1152876

**Published:** 2023-10-19

**Authors:** Alessia Raffagnato, Marina Miscioscia, Gaia Bruni, Lara Del Col, Annalisa Traverso, Martina Ferrarese, Caterina Ancora, Silvia Zanato, Michela Gatta

**Affiliations:** ^1^Child and Adolescent Neuropsychiatric Unit, Department of Women's and Children's Health, University Hospital of Padua, Padova, Italy; ^2^Department of Developmental Psychology and Socialisation, University of Padua, Padova, Italy

**Keywords:** COVID-19, pediatric age, mental health, neuropsychiatry, hospitalization, readmission

## Abstract

**Purpose:**

This study aimed to investigate the specific risk factors and psycho-social and clinical features of hospitalized neuropsychiatric patients during the COVID pandemic and to analyze the hospital readmission phenomenon, which, according to recent studies, increased in frequency during the first pandemic period.

**Patients and methods:**

This observational retrospective cohort study examined 375 patients aged between 0 and 17 years who were hospitalized between 1 February 2018 and 31 March 2022 due to neuropsychiatric issues. The majority of the patients were girls: there were 265 girls compared to 110 boys (*M* = 13.9 years; SD 2.30 years). The total sample was divided into two groups: the *pre-COVID-19 group* (160 inpatients hospitalized between February 2018 and February 2020) and *the COVID-19 group* (215 inpatients hospitalized between March 2020 and March 2022). To explore the readmission phenomenon (second aim), we selected from the two groups of patients with at least one hospital readmission within 365 days after the first discharge. Multiple variables (sociodemographic, clinical, psychological, and related to hospitalization) were collected for each patient by reviewing their medical records.

**Results:**

The risk factors for mental health disorders were similar between the two groups, except for the significantly increased use of electronic devices in the COVID-19 group, increasing from 8.8% in the pre-COVID-19 group to 29.2% in the COVID-19 group. Patients suffering from eating disorders increased from 11.3% in the pre-COVID-19 group to 23.8% in the COVID-19 group. Hospital readmissions nearly increased from 16.7% in the 2-year pre-COVID-19 period to 26.2% in the 2-year COVID-19 period. A total of 75% of patients hospitalized three or more times in the last 2 years and 85.7% of the so-called “revolving door” patients (with relapse within 3 months after discharge) were identified in the COVID-19 group. However, the comparison between the two groups of patients readmitted before and during the COVID-19 pandemic did not show any differences in terms of sociodemographic and clinical characteristics.

**Conclusion:**

In conclusion, there was a significant increase in hospital readmissions, but these results suggest the need for better coordination between hospital and territorial services in managing the complexity of mental health problems related to situations arising from the COVID-19 pandemic and the necessity to implement prevention strategies and services.

## 1. Introduction

At the beginning of 2020, the whole world was forced to deal with the COVID-19 pandemic and the subsequent security measures, which seemed to be the main source of psychological stress among children and adolescents (1), who reported higher levels of isolation and loneliness during the government lockdowns (2). These factors probably led to an increase in research on ways to connect with peers, resulting in the higher usage of technological devices and social media and a subsequent decrease in sports activities (3). The COVID-19 pandemic has strongly affected children's and adolescents' mental health, with increases in symptoms of anxiety and depression, internalizing and externalizing psychopathology, and stressful environmental situations (1–6). Additionally, based on their gender and age, studies suggest that youths reacted differently to the COVID-19 pandemic (1, 5, 7, 8). Regarding referrals to mental health services, national and international literature corroborates detecting different trends according to different phases of the pandemic: a “freezing” of emergency psychiatric visits during the first pandemic period, followed by increased use of emergency services and stabilization at higher levels in the following summer months (9–11). It was found that, despite a decline in emergency department presentations during the first lockdown, a greater proportion of assessed children and adolescents met the admission criteria compared to the previous year, with an increase in the acuity of mental health disorders (11, 12).

Considering that the scientific literature documents a constant increase in psychiatric hospitalizations of youths over the last decade and that, even before the outbreak of the COVID-19 pandemic (13–16), there was already a significant shortage of beds in child neuropsychiatry units (compared to the estimated needs in the pre-pandemic period) in Italy, the COVID-19 pandemic caused a breakdown in mental health inpatient and outpatient services. However, quite a few studies have investigated the effect of the COVID-19 pandemic on mental health-related hospital admissions among youths in the 2 years since the pandemic first began.

In light of the daily clinical and nursing experience of the past 3 years, which has shown an increase in psychopathological complexity and some clinical pictures, such as self-harm (17) and eating disorders (18), we aimed to identify eventual specific risk factors and psycho-social and clinical features of young persons with psychiatric diseases to better target diagnostic and therapeutic interventions. To reach our goal (aim 1), we analyzed the psycho-social and clinical variables of hospitalized neuropsychiatric patients during the COVID-19 pandemic, particularly the phenomenon of hospital readmission (aim 2). The latter is considered an indicator of clinical severity and greater complexity in case management. This phenomenon appears to have increased in frequency during the early pandemic period, according to some local and international studies (19, 20) recording an unprecedented total number of rehospitalizations/patients. We expected to find a greater impairment of the inpatient population during the COVID period in terms of personal and environmental risk factors, psychopathological severity, and management complexity during hospitalization.

## 2. Material and methods

### 2.1. Procedures and participants

We conducted an observational retrospective cohort study by reviewing medical materials (clinical records, medical examination reports, neuropsychiatric interviews with patients and their parents, discharge reports, and clinical reports sent to territorial mental health and social services). The data were treated anonymously and in accordance with the guidelines of the Declaration of Helsinki 2013. This study was approved by the Institutional Ethics Committee of University Hospital of Padua (CESC Prot. N. 0044914, 13.07.2021).

With regard to the first study aim, two groups of neuropsychiatric inpatients were enrolled. The inclusion criteria for both groups were children and young adults who were aged between 0 and 17 years and had been hospitalized for more than 24 h in the Child and Adolescent Neuropsychiatric Unit, University Hospital of Padua between 1 February 2018 and 31 March 2022. Outpatients admitted to neuropsychiatric visits, inpatients with 1 day hospital stay, or those who prematurely self-discharged before 24 h were excluded from the study. In total, the number of patients enrolled was 375. The majority of patients were girls: 265 girls vs. 110 boys. The mean age was 13.9 years (SD 2.30 years). For study purposes, the total sample was divided into two groups: the *pre-COVID-19 group*, which included 160 inpatients hospitalized between February 2018 and February 2020, and *the COVID-19 group*, which enrolled 215 inpatients hospitalized between March 2020 and March 2022. To explore the phenomenon of readmission (second aim), we selected participants from the two groups of patients with at least one hospital readmission within 365 days after the first discharge.

**Figure 1:**
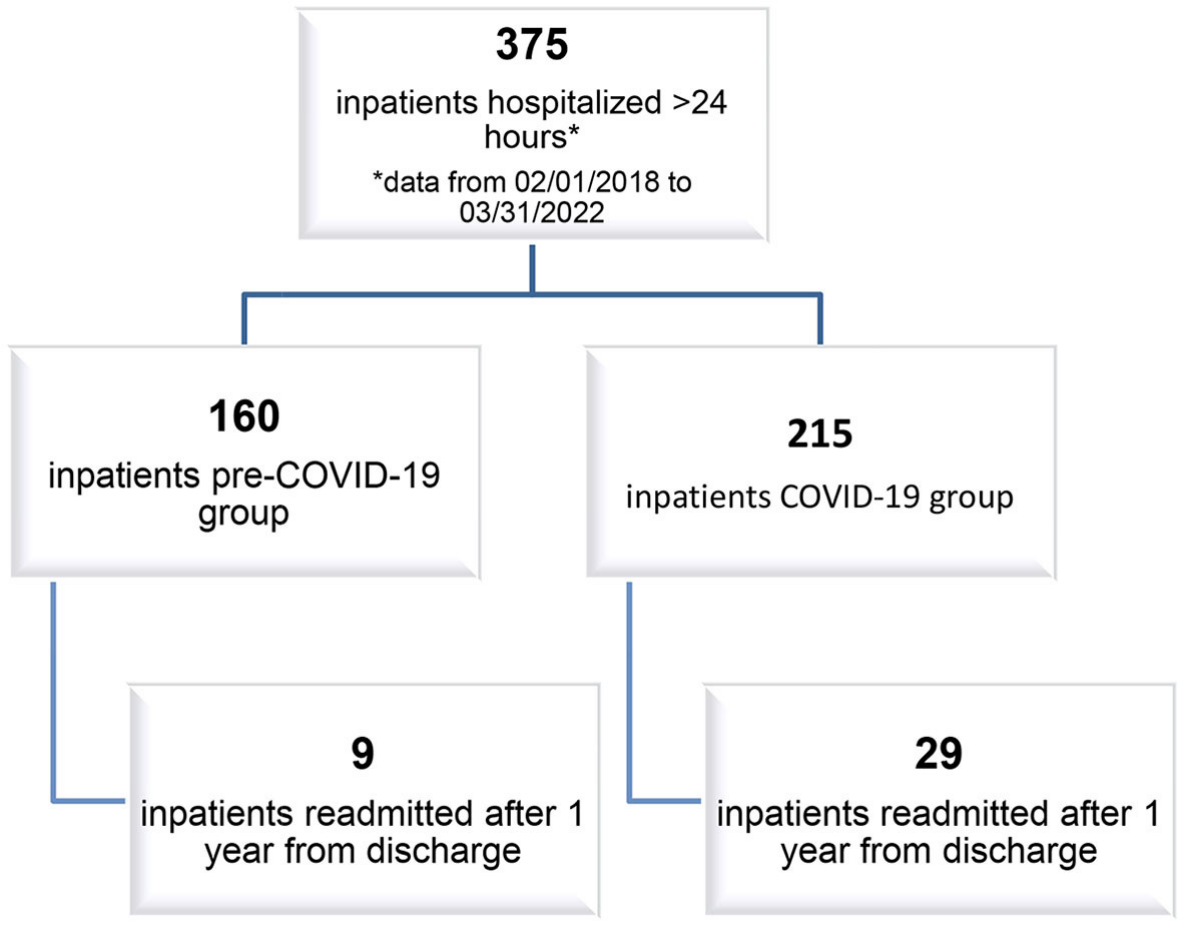
Flowchart.

In total, we included 38 patients, nine of whom belonged to the “pre-COVID-19 Hospital Readmission (HR) group,” with an average age of 14.3 years (SD 1.66 years), and 29 of whom belonged to the “COVID-19 HR group,” with an average age of 14.4 years (SD 1.76 years). Considering gender, we enrolled 30 female and eight male patients. The two samples of relapsing patients were compared based on risk factors (well-known in the literature) linked to hospital readmission in a psychiatric ward to investigate possible sociodemographic and clinical differences. Figure 1 shows the flowchart of the two study aims.

### 2.2. Data collection

A Microsoft Excel database was created and scrupulously compiled to collect data. Multiple variables, which are described below, were collected for each patient: sociodemographic variables—gender (girls vs. boys), age at the time of hospitalization (quantitative data collected), ethnicity (Caucasian, Latin, African, Asian, or “other” when parents belong to different ethnicities), history of immigration to Italy (yes or no), educational level (primary, middle, or high school), academic or school behavioral problems (yes or no), peer socialization (good, difficult, or social withdrawal), and bullying/cyberbullying (yes or no). From the collection of family anamnesis, information was obtained regarding the number of siblings (single child, one sibling, ≥two siblings), the marital status of parents (married/separated/widowed/other), familiarity with psychiatric disorders (yes or no), other health problems in family members (yes or no), and intra-family conflict (yes or no). The collected clinical anamnesis variables were the presence of chronic diseases (yes or no), traumatic life events (yes or no), previous requests for psychological assistance or admittance to neuropsychiatric services (yes or no), high-risk behavior such as alcohol use (yes or no) and substance use (yes or no), and the usage of technological devices (more or <4 h per day); suicidal self-injury: suicidal ideation (SI; yes or no), suicide attempt (SA; yes or no), and method of suicide attempt, such as drug or substance poisoning/self-cutting/other; non-suicidal self-injury (NSSI; yes or no), frequency of self-harming acts (more or less than five times per year in accordance with DSM-5 criteria), self-injured body parts (one or more than one), and age of the first self-injurious act (quantitative data); and eating problems: focus on food and body image (yes or no), diagnoses of eating disorders (anorexia nervosa, bulimia nervosa, or binge-eating disorder), and age of onset. Variables related to hospitalization were the reason for hospitalization (suicidal and non-suicidal self-injury, anxiety symptoms and/or functional symptoms, eating disorders, psychomotor agitation and/or aggression, psychotic symptoms, and other); hospitalization modality (urgent or scheduled), method of hospitalization access [Emergency Department (ED); neuropsychiatric consulting, outpatient service, scheduled hospitalization, and transfer from another medical unit or from another hospital]; length in days of the hospitalization; post-discharge relapse (yes/no and number of readmission to the hospital); diagnosis according to ICD-10 criteria (psychotic disorders F20–29, affective syndromes F30–39, neurotic stress-related and somatoform disorders F40–48, syndromes and disorders associated with abnormal physiological functions F50–59, behavioral and emotional syndromes and disorders with onset in childhood and adolescence, and personality disorders F90–98/F60 or other F70/F80/Z); psychiatric comorbidity (yes or no); pharmacological therapy (monotherapy or polytherapy and pharmacological combination: neuroleptics, antidepressants, benzodiazepines, and mood stabilizers); and post-discharge services (public and private territorial outpatient care, residential or semi-residential care, intensive hospital monitoring, social services, or family counseling). Additional variables were collected only for patients with at least one hospital readmission: readmission time (in days), defined as the difference between the date of index discharge and date of second rehospitalization; the presence of relapse within 1 year after discharge and the first 3 months after discharge (revolving door); and the length in days of the second hospitalization.

### 2.3. Statistical analysis

Data were analyzed using Jamovi Statistic Software (the Jamovi project, version 1.6.23.0). The statistical significance level was set at a *p*-value of ≤ 0.05. Mean, standard deviation (SD), and frequency were used for conducting a descriptive analysis. Contingency tables and the chi-squared test were used for the categorical variables to test for the distribution in terms of gender, psychiatric familiarity, school problems, psychiatric comorbidity, SI, SA, NSSI, and relapse within 1 year and within 3 months from discharge to highlight the risk factors and psycho-social and clinical features of hospitalized young patients in the two groups. After verifying the validity of the assumptions, we performed a *t*-test for independent samples. The test aimed to identify differences between the two groups in several continuous variables. These variables included the age at which the first self-injurious act occurred, the age of onset for eating disorders, the duration of the second hospitalization in days, and the number of readmission times.

## 3. Results

### 3.1. Demographic, socio-familial, and academic variables

The results of demographic, socio-familiar, and educational variables are shown in Table 1.

**Table 1:** Socio-demographic, socio-family and academic variables.

**Socio-demographic variables** ***n*** **(%)**		**Pre-COVID-19 (*n* = 160)**	**COVID-19 (*n* = 215)**	***p*-value**
Sex	Male	51 (31.9%)	59 (27.4%)	0.351
	Female	109 (68.1%)	156 (72.6%)	
Average age (SD)		13.5 years (SD 2.56 years)	14.3 years (SD 2.03 years)	
Ethnicity	Caucasian	140 (89.2%)	190 (89.2%)	0.184
	Latin	4 (2.5%)	0 (0.0%)	
	African	6 (3.8%)	12 (5.6%)	
	Asian	3 (1.9%)	4 (1.9%)	
	Other	4 (2.5%)	7 (3.3%)	
Past history of immigration to Italy	Presence	19 (12.0%)	11 (5.1%)	0.016
Marital status of parents	Married/cohabiting parents	105 (67.3%)	147 (68.4%)	0.987
	Divorced parents	41 (26.3%)	55 (25.6%)	
	Widowed parent	4 (2.6%)	6 (2.8%)	
	Other	6 (3.8%)	7 (3.3%)	
Intra-family problems	Presence	77 (50.0%)	98 (47.8%)	0.680
Siblings	1 sibling	86 (54.8%)	113 (53.1%)	0.911
	≥2 siblings	27 (17.2%)	39 (18.3%)	
	Only child	29 (18.5%)	39 (18.3%)	
Psychiatric familiarity	Presence	90 (58.8%)	141 (66.2%)	0.149
Others family health problems	Presence	95 (60.9%)	138 (64.8%)	0.444
Educational level	High school	72 (46.8%)	131 (60.9%)	0.014
	Middle school	58 (37.7%)	67 (31.2%)	
	Primary school	22 (14.3%)	17 (7.9%)	
School problems	Presence	97 (62.6%)	126 (59.4%)	0.542
Bullying	Presence	40 (25.6%)	60 (28.3%)	0.571

SD, standard deviation; Y, years.

In the two periods, female patients used neuropsychiatric services more than male patients.

Although there were no differences in ethnicity, those with a history of immigration comprised 12.0% of the pre-COVID-19 group and 5.1% of the COVID-19 group [χ^2^ (1, *N* = 372) = 5.81, *p* = 0.016, a decrease of 7%, 95% CI (1%, 13%)]. There were no differences between the two periods regarding socio-family and school variables, except for educational level [primary, middle, and secondary school; χ^2^ (3, *N* = 369) = 10.6, *p* = 0.014].

### 3.2. Anamnesis-clinical variables and hospitalization-related variables

Regarding the anamnesis data, there were no significant changes when comparing the two periods. A total of 48.4% of patients in the pre-COVID-19 group and 41.1% of those included in the COVID-19 group suffered from one or more chronic diseases (asthma, allergies, diabetes mellitus, IBD, etc.). Over 80% of inpatients in both groups had previously accessed neuropsychiatric (NPI) services or received other types of support (psychological, psychotherapeutic, or psychiatric). Almost half of the patients (44%) of both groups experienced at least one traumatic event, such as parental divorce, family mourning, or a scholarly failure. As shown in Table 2, a statistically significant increase [χ^2^ (1, *N* = 368) = 23.1, *p* < 0.001, an increase of 10%, 95% CI (13%, 28%)] was found by comparing the use of electronic devices (more than 4 h per day: from 8.8% in the pre-COVID-19 group to 29.2% in the COVID-19 group). The percentages more than tripled in the pandemic period, showing how the pandemic significantly affected young people's lifestyles from this point of view.

**Table 2:** Use of alcohol, psychotropic substances, tobacco, and electronic devices.

	**Pre-COVID-19 (*n* = 160)**	**COVID-19 (*n* = 215)**	***p*-value**
Use or abuse alcohol *n* (%)	10 (6.3%)	15 (7.1%)	0.759
Use or abuse substances *n* (%)	9 (5.7%)	13 (6.2%)	0.852
Use or abuse tobacco *n* (%)	10 (6.4%)	21 (10.0%)	0.221
Use or abuse electronic devices (>4 h a day) *n* (%)	14 (8.8%)	61 (29.2%)	< 0.001

### 3.3. Modality, reason for hospitalization, and diagnosis

Regarding the modalities of hospitalization, whether planned or compulsory, the results were uniform in the two periods (planned hospitalizations reached 14.5% in the pre-COVID-19 period vs. 14.1% in the COVID-19 period, while urgent hospitalizations reached 85.5% in the pre-COVID-19 period vs. 85.9% in the COVID-19 period). There was an increase in admissions from the Emergency Department following a visit to the hospital's outpatient service.

The reasons for hospitalizations are reported in Table 3, showing increased numbers of patients with eating disorders, suicidal and non-suicidal self-injury, and psychomotor agitation/aggression.

**Table 3:** Total percentage frequency of reasons for hospitalization.

		**Pre COVID-19 (*n* = 160)**	**COVID-19 (*n* = 215)**	***p*-value**
Reasons for hospitalization	Suicidal and non-suicidal self-injury	51 (32.5%)	71 (33.6%)	0.206
	Anxiety and/or functional symptoms	22 (14%)	22 (10.4%)	
	Eating disorder	26 (16.6%)	49 (23.2%)	
	Psychomotor agitation/aggression	24 (15.3%)	39 (18.5%)	
	Psychotic symptoms	11 (7%)	13 (6.2%)	
	Other	23 (14.6%)	17 (8.1%)	

Data on the first and second ICD-10 (International Classification of Disease System) diagnoses at discharge are shown in Table 4. A total of 17 inpatients did not receive a complete diagnosis (early discharge due to the parents' choice or transfer to another medical unit).

**Table 4:** Total percentage frequency of first and second ICD-10 diagnosis.

**Diagnosis** ***n*** **(%)**		**Pre-COVID-19 (*n* = 160)**	**COVID-19 (*n* = 215)**	***p*-value**
First diagnoses	Affective syndrome (F30–39)	55 (36.7%)	73 (35.1%)	0.103
	Neurotic, stress-related, and somatoform disorders (F40–48)	45 (30.0%)	51 (24.5%)	
	Disorders associated with physiological disturbances (F50–59)	16 (10.7%)	48 (22.6%)	
	Personality disorders, syndromes, and behavioral disorders with onset usually occurring in childhood/adolescence (F60, F90–98)	17 (11.3%)	19 (9.1%)	
	Psychotic disorders (F20–29)	11 (7.3%)	12 (5.8%)	
	Other (F 70/80, Z)	6 (4.0%)	6 (3.4%)	
Second diagnosis	Affective syndrome (F30–39)	14 (14.4%)	31 (19.4%)	0.096
	Neurotic, stress-related, and somatoform disorders (F40–48)	34 (35.1%)	72 (45.0%)	
	Disorders associated with physiological disturbances (F50–59)	4 (4.1%)	6 (3.8%)	
	Personality disorders, syndromes, and behavioral disorders with onset usually occurring in childhood/adolescence (F60, F90–98)	19 (19.6%)	31 (19.4%)	
	Psychotic disorders (F20–29)	3 (3.1%)	3 (1.9%)	
	Other (F 70/80, Z)	23 (23.7%)	17 (10.6%)	

Between the two periods, there was a decline in all diagnoses, except for ICD-10 F50–59 codes, which include eating disorders. Nearly 75% of patients with these diagnoses were hospitalized during the 2-year pandemic period, compared with only 25% of patients during the 2-year pre-pandemic period. With regard to comorbidity (the presence of two or more psychiatric diagnoses in the same patient), we observed a statistically significant increase from 66.4% of inpatients in the pre-pandemic period to 77.0% in the pandemic period [χ^2^ (1, *N* = 358) = 4.91, *p* = 0.027, increase of 11%, 95% CI (1%, 20%)].

Hospitalization lasted an average of 20.8 days (SD 19.0) in the pre-COVID-19 period and 19.9 days (SD 16.3) in the COVID-19 period.

### 3.4. Suicidal and non-suicidal self-injury

With regard to suicidality, no significant results were found; from a descriptive point of view, small increases in suicidal ideation (from 44.0 to 53.1% between the two periods) and in suicide attempts (from 22.0 to 27.1%) were detected. Suicidal methods changed: in the COVID-19 period, we registered increasing suicidal attempts through drugs or substance poisoning (from 44.7 to 56.7%) but not through wrist cutting, whose percentages were comparable (7.9% pre-COVID-19 and 7.3% COVID-19). Suicidal methods classified as “other” (defenestration, falling from heights, choking, and being hit by fast vehicles) dropped from 47.4 to 36.4%. With regard to NSSI, the percentage of NSSI inpatients was 40.6% in the pre-COVID-19 period and 44.2% in the COVID-19 period. Meanwhile, for NSSI frequency acts, 52.3% of inpatients had an occasional history of NSSI in the pre-COVID-19 period (less than five acts per year), compared to 62.2% of inpatients in the COVID-19 period. In comparison, 37.8 vs. 47.7% of inpatients had a history of repetitive NSSI (more than five times a year), respectively, in the two periods. Regarding the number of self-injured body parts, in the pre-COVID-19 period, most inpatients self-injured multiple body parts (vs. self-injuring a single body part), 58.1 vs. 41.9%, respectively; during the 2-year COVID-19 period, there was a reversal of the trend: 31.2% of inpatients self-injured multiple body parts, while 68.8% of inpatients self-injured a single body part. This result had statistical significance [χ^2^ (1, *N* = 136) = 8.93, *p* = 0.003, an increase of 27%, 95% CI (−44%, −10%)].

Statistically significant changes [χ^2^ (4, *N* = 148) = 11.7, *p* = 0.019] in the reasons for self-injuring acts (self-punishment, relief from negative emotions, both known and unknown) have been observed. Specifically, the reason “relief from suffering” both alone and grouped with “self-punishment” increased (from 40 to 53.8% and from 1.8 to 10.8%, respectively). Moreover, we found a significant increase [*t*_(127)_ = –3.90; *p* < 0.001] in the age of the first self-injurious act from an average age of 12.7 years (SD 1, 41 years) in the pre-pandemic period to an average age of 13.8 years (SD 1.43 years) in the pandemic period.

### 3.5. Eating disorders

Regarding patients with eating disorders, particularly anorexia nervosa, we registered three times more patients with anorexia nervosa in the COVID-19 period than in the pre-COVID-19 period; respectively, the percentages were 73.9 vs. 23.8%. The increase related to eating problems had statistical significance [χ^2^ (1, *N* = 374) = 9.63, *p* = 0.002, increase of 13%, 95% CI (5%, 20%)], with an increase from 11.3% in the pre-COVID-19 group to 23.8% in the COVID-19 group. We observed a significant increase [*t*_(67)_ = 3.59; *p* < 0.001] in the age of the onset of eating disorders from the pre-pandemic period (a mean age of 12.6 years) to the pandemic period (a mean age of 14.1 years).

### 3.6. Post-relapse discharge

**Figure 2:**
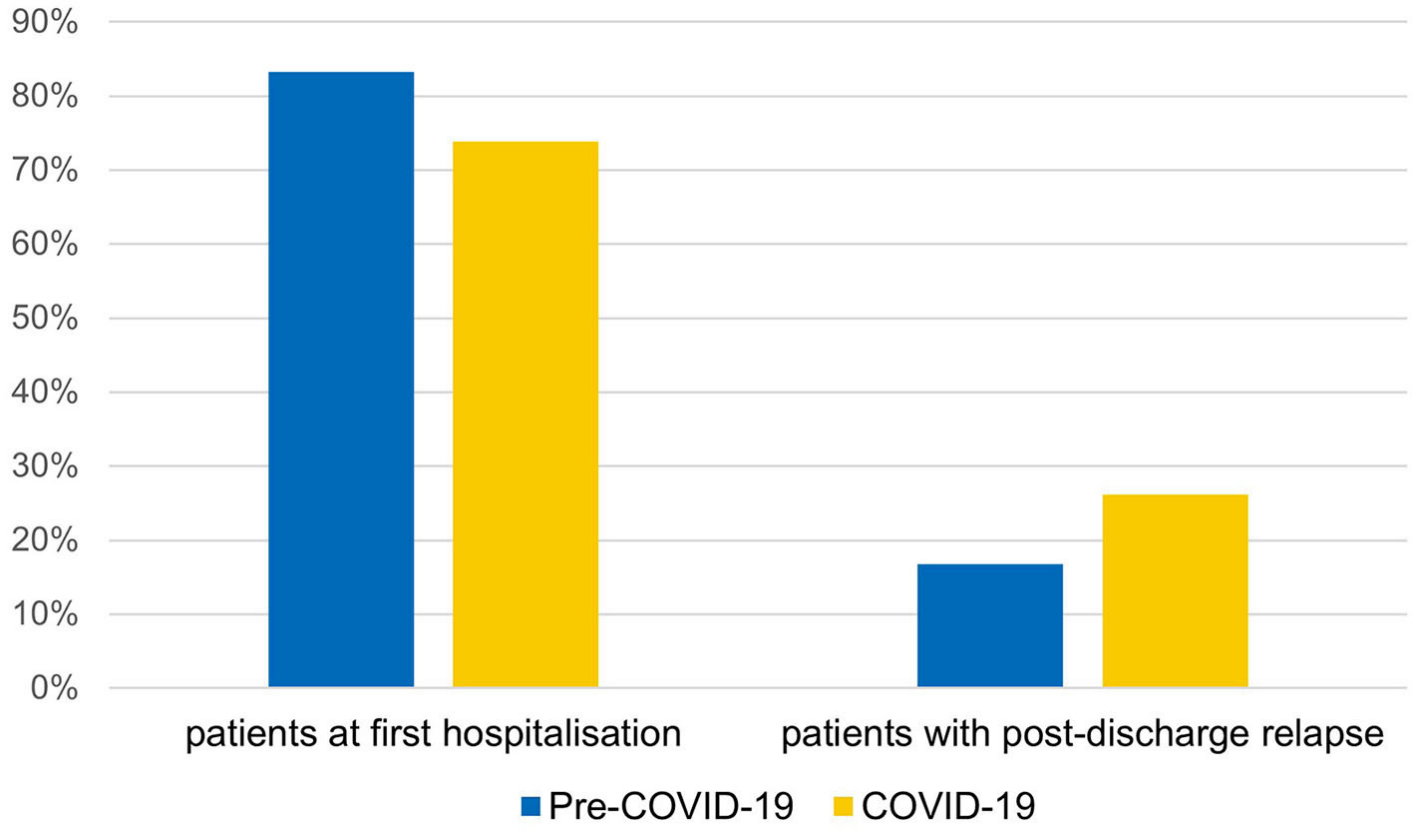
Percentages of patients with post-discharge relapse between the two periods.

Data regarding post-discharge hospital readmission showed that, in the pre-pandemic period, relapses reached 16.7%, while in the pandemic period, this percentage increased to 26.2, meaning that, in the pandemic period, compared to the pre-pandemic period, more inpatients had already been hospitalized at least once (Figure 2). This increase had statistical significance [χ^2^ (1, *N* = 304) = 3.92, *p* = 0.048; an increase of 10%, CI (0%, 19%)], from 16.7% in the 2-year pre-COVID-19 period to 26.2% in the COVID-19 period.

Considering the number of total hospitalizations per patient, 75% of patients hospitalized three or more times were part of the COVID-19 group. This percentage in the COVID-19 period also included patients hospitalized up to five, six, or seven times; these numbers were never reached by the pre-COVID-19 group. Furthermore, patients with at least one readmission within 1 year after discharge reached 76.3% in the COVID-19 group and only 23.7% in the pre-COVID-19 group [χ^2^ = 6.73, df = 1, *p* = 0.009, an increase of 11%, 95% CI (3%, 18%)]. More than half (55.3%) of these patients were “revolving doors,” as hospital readmission occurred within 3 months after discharge. Since these data showed that the phenomenon of hospital readmissions (HR) was much more frequent in the pandemic period than in the pre-pandemic one, it was considered appropriate to carry out further analyses only on patients affected by this phenomenon. It was found that the “pre-COVID-19 HR group” and the “COVID-19 HR group” did not differ significantly with regard to the following: gender (~78% girls and 22% boys in both groups), the presence of psychiatric familiarity (66.7 vs. 58.6%), school problems (88.9 vs. 62.1%), psychiatric comorbidity (77.8 vs. 72.4%), SI (88.9 vs. 72.4%), SA (33.3 vs. 31.0%), NSSI (66.7 vs. 62.1%), and revolving door nature, i.e., readmission within 3 months after discharge (33.3 vs. 62.1%). Interestingly, 85.7% of “revolving door” patients were part of the COVID-19 HR group. Regarding the readmission time, the average number of days before the second hospitalization was considerably lower for the COVID-19 HR group, 97 days (SD 105.0), compared to the pre-COVID-19 HR group, 157 days (SD 93.1).

The average length of the second hospitalization of relapsing patients was 25.3 days (SD 23.5) in the pre-COVID-19 HR group and 19.2 days (SD 15.5) in the COVID-19 HR group.

## 4. Discussion

This study investigated the phenomenon of NPI admissions during the COVID-19 pandemic, comparing them with admissions in the immediately preceding period, focusing on the psycho-social risk factors connected with hospitalization and on readmissions during the first year after discharge, in regard to which there is little data in the literature, to improve the diagnostic-therapeutic care of adolescents with psychiatric problems.

### 4.1. Educational level

Compared to the pre-COVID-19 group, it is possible that the excess share of patients during the COVID-19 pandemic were those who were exclusively attending high school. Consistent with the literature on the subject (21), during the pandemic period, the severity of psychopathology appeared to be higher in middle school students than in primary school students, and it was the highest in high school students. Indeed, considering the average age of inpatients in the pandemic 2-year period, i.e., 14.3 years, it is coherently rather plausible that a good percentage of them were experiencing a school transition period (secondary school), which implies changes in habits and the relational environment. These changes can be added up and contextualized into those of adolescence, including physical and hormonal ones, as well as those related to the processes of one's own identity and self-efficacy. Additionally, the long or short suspension of face-to-face teaching and the quarantine may have hindered teenagers' achievement of a new balance or even deprived them of stable peer relationships in the school environment, which are important protective factors from the onset of mental health disorders (22).

### 4.2. Substance abuse

With regard to lifestyles, the increasing trends in the consumption of alcohol, psychotropic substances, and tobacco in the COVID group already observed in Gatta et al.'s study (19) 12 months after the beginning of the pandemic were also confirmed 24 months after its start. In line with this, the longitudinal study by Dumas et al. (23) tracked alcohol and cannabis consumption during alternating periods of confinement and re-release for a total of 14 months, showing a gradually increasing use of these substances.

### 4.3. Technologic devices

Our study investigated the role of devices and, thus, social networks in the context of psychopathological disorders in hospitalized patients. Electronic device abuse is undoubtedly one of the main consequences of the deleterious effects of COVID-19 pandemic measures on children's mental health. Children and adolescents confined at home have attempted to alleviate negative feelings and experiences related to the pandemic period by spending more time online. However, if media use can be identified as a coping strategy and a protective factor for children's mental health during the pandemic, then it is also an important risk factor. The association between excessive Internet use (more than 3 h per day) and internalizing mental problems, particularly anxiety and depression, is well known (24). We would especially like to highlight the risk of developing body image, diet, and exercise concerns associated with increased social media use (25). This may have contributed to the increase in the number of eating disorders recorded during the pandemic. A previous study (19) found no significant differences in the increased use of electronic devices during the first year of the pandemic compared to the previous one.

Conversely, 2 years after the pandemic outbreak, the percentage of children using these devices for more than 4 h/day quadrupled. This suggests that, despite the weakening of isolation measures as the pandemic progressed, electronic devices remained a habit and the primary means of social connection and spending time. A study conducted in Ferrara on a sample of Italian children and adolescents showed that, after the second pandemic wave, not only did the number of children with smartphone addiction increase but so did the number of children at greater risk of addiction compared to before the pandemic (26). When studying why our patients come to the hospital, we found that, compared to the 2 years before COVID-19, a higher number of patients experienced suicidal ideation and attempted suicide.

### 4.4. Self-injury

Despite what was expected and the data in the literature, our study did not reveal an increase in suicidal or non-suicidal self-harm as an incidence of the phenomenon, but rather, differences were found in relation to features of the NSSI in terms of the number of self-injured body parts, which decreased during the pandemic period, and in the reasons for self-injuring acts, with predominantly relief from negative and blaming emotions. Moreover, we found a significant increase in the age of the first self-injury act during the pandemic period, which might suggest that the onset of the disorder was recent in young self-injurers of the pandemic period compared with pre-pandemic young self-injurers. With regard to the above results, one of the possible explanations is that young people were more often under adult control due to periods of confinement.

### 4.5. Eating disorders

Finally, the statistically significant increase in patients with eating disorders during the pandemic is consistent with the international and national literature. A Canadian study reported that ED visits and hospitalizations of pediatric patients suffering from these disorders increased significantly (by 66 and 37%, respectively) as soon as COVID-19 started, compared to the expected rates before the pandemic, and remained well above the expected levels during the first 10 months of the pandemic. Specifically, hospitalizations of adolescents aged 14–17 years were significantly higher than expected, in line with our results (27). Particularly, with regard to anorexia nervosa, the COVID-19 pandemic seems to have had a deep impact in terms of symptom severity, which is mirrored by a large increase in admission rates across Europe (20, 28).

### 4.6. Multiple diagnoses

It is also important to underline that, in agreement with the increase in severity and clinical complexity, independent of the psychiatric diagnosis, the number of patients with at least one psychiatric comorbidity increased over the 2 pandemic years (from 66.7 to 77%).

### 4.7. Post-discharge relapses

It is striking how, within this phenomenon, cases with three or more close hospitalizations after the first admission increased. However, the comparison between the two groups of patients rehospitalized before and during the COVID-19 pandemic did not reveal any differences in sociodemographic (gender, age, the presence of psychiatric familiarity, and school problems) and clinical (the presence of SI, NSSI, previous SA, and psychiatric comorbidity) characteristics. This suggests that this phenomenon mainly depended on the inability of territorial services to ensure adequate patient care after hospital discharge, together with increased severity and clinical complexity (12, 20). With regard to the findings, we observed an increase in comorbidities related to eating disorders during the COVID-19 period. There are probably no specific services equipped to handle such a large influx of patients. Additionally, there was a rise in admissions among adolescents (high school students), particularly high school students. This could align with the notion that a delicate transition period is needed involving both child neuropsychiatric and adult psychiatric services. Furthermore, the reorganization of services due to the COVID-19 pandemic meant reduced access to in-person treatment, restricted entry into intensive psychiatric treatment programs, the stopping of services such as group therapies and daily centers, and prolonged wait times for those seeking to establish care. Further confirmation of the above considerations would lie in the significant increase in the use of pharmacotherapy during the 2-year pandemic period, specifically neuroleptic associations with other pharmaceuticals.

This study has some limitations, such as patients' heterogeneity in age and other correlated variables. Moreover, the SARS-CoV-2 sanitary infection is still ongoing, although not in a state of emergency, and it is not yet possible to have a complete overview of its effects.

## 5. Conclusion

Given the results of this study, we can conclude that the 2 years after the pandemic outbreak, adolescents, especially high school students, have been the most affected. The increased use of electronic devices, a known risk factor for mental health, stands out as one of the main consequences of the pandemic period on the lifestyles of adolescents (29). The increase in hospital readmissions, without any significant pre/post-pandemic differences in terms of sociodemographic and clinical characteristics of relapsed patients, prompts consideration of better coordination between hospital and territorial services to prevent the need for hospitalization and contain relapses requiring rehospitalization. Certainly, further research, hopefully polycentric, is needed to better delineate the roots of this phenomenon. The consequences arising from the SARS-CoV-2 health emergency undoubtedly highlight the weakness of the resources needed to provide primary and secondary prevention measures to protect the mental health of tomorrow's adults.

## Data availability statement

The raw data supporting the conclusions of this article will be made available by the authors, without undue reservation.

## Ethics statement

The studies involving humans were approved by Institutional Ethics Committee of University Hospital of Padua (CESC Prot. N. 0044914, 13.07.2021). The studies were conducted in accordance with the local legislation and institutional requirements. Written informed consent for participation in this study was provided by the participants' legal guardians/next of kin.

## Author contributions

Conceptualization, methodology, funding acquisition, and supervision: MG. Formal analysis: MM and AR. Data curation: AR and GB. Investigation: CA, AT, SZ, and LD. Writing—original draft preparation: AR, GB, and MG. Writing—review and editing: AR, MM, GB, MF, and MG. All authors have read and agreed to the published version of the manuscript.
